# Protein kinase G type-I phosphorylates c-Src at serine-17 and promotes cell survival, proliferation and attachment in human mesothelioma and non-small cell lung cancer cells

**DOI:** 10.1186/1471-2210-11-S1-O31

**Published:** 2011-08-01

**Authors:** Ronald R Fiscus, Mary G Johlfs

**Affiliations:** 1 Center for Diabetes & Obesity Prevention, Treatment, Research & Education, and the College of Pharmacy, Roseman University of Health Sciences (formerly the University of Southern Nevada), Henderson, Nevada, USA

## Background

Previously, we demonstrated that protein kinase G type-Iα (PKG-Iα) plays an important role in promoting cell survival in neural cells (N1E-115 neuroblastoma and NG108-15 neuroblastoma-glioma hybrid cells) and significantly contributes to the serine-155 phosphorylation of BAD, an apoptosis-regulating protein [1]. We also found that PKG-Iα promotes cell survival and proliferation in mouse OP9 bone marrow stromal cells [2] and human ovarian cancer cells [3] [determined by using both pharmacological inhibitors (ODQ, DT-2 and DT-3) and gene knockdown (siRNA) to reduce PKG-Iα/β activity]. In the case of ovarian cancer cells, which predominantly express the PKG-Iα isoform, the pro-growth and pro-survival effects involved a novel interaction between PKG-Iα and the oncogenic protein c-Src [3]. For example, intracellular activation of PKG-Iα, assessed by VASP serine-239 phosphorylation, was dependent on c-Src-catalyzed tyrosine phosphorylation of PKG-Iα (i.e. VASP phosphorylation was blocked by Src inhibitors, SKI-1 or SU6656) and the intracellular activation of c-Src was (somehow) dependent on the kinase activity and expression levels of PKG-Iα (i.e. c-Src activation was decreased by DT-2 or siRNA-induced knockdown of PKG-Iα).

## Hypothesis, research plan and methods

We hypothesize that c-Src activation in cancer cells involves the PKG-Iα-catalyzed phosphorylation of a serine or threonine residue of c-Src, likely serine-17 (because amino acid residues around the serine-17 site of c-Src provide a good consensus sequence for PKG-Iα/β). The present study determines: 1) if PKG-Iα/β can directly phosphorylate c-Src at serine-17 using *in vitro* conditions, 2) if serine-17-phophorylation of c-Src, in intact cancer cells, is dependent on PKG-Iα/β expression and kinase activity (using pharmacological inhibitors and shRNA gene knockdown), and 3) if the same inhibition/knockdown of PKG-Iα/β alters cell survival, proliferation and attachment. Objectives # 2 and 3 were tested in a non-small cell lung cancer (NSCLC) cell line, NCI-H23, and two mesothelioma cell lines, MSTO-211H and NCI-H2052. The serine-17 phosphorylation of c-Src was determined by Western blot analysis using an antibody (developed in collaboration with Cell Signaling Technologies) that is specific for the phosphorylated-serine-17 site of c-Src. Protein expression of PKG-Iα and PKG-Iβ was determined by both conventional Western blot analysis and a new ultrasensitive, highly-quantitative capillary-electrophoresis-based immuno-quantification system, the NanoPro100 system (Cell Biosciences, Inc., Santa Clara, CA, USA). The NanoPro100 system allows clear separation and identification of the different PKG-I isoforms and phosphorylated forms.

## Results

In *in vitro* incubations using recombinant human c-Src and either PKG-Iα or PKG-Iβ, both recombinant PKG-Iα and PKG-Iβ were able to directly catalyze the phosphorylation of serine-17 in c-Src, resulting in increased autophosphorylation of c-Src at tyrosine-419 (equivalent to tyrosine-416 in mouse c-Src). The NSCLC cells (NCI-H23 lung cancer cells) were found to express only the PKG-Iα isoform, whereas both of the mesothelioma cell lines (MSTO-211H and NCI-H2052) express both PKG-Iα and PKG-Iβ. In all cell lines, inhibition of PKG-Iα/β kinase activity using DT-2 or silencing of PKG-Iα/β expression using shRNA dramatically reduced the intracellular phosphorylation of c-Src at serine-17, whereas addition of the PKG activator 8-bromo-cGMP increased c-Src phosphorylation at serine-17. Both the kinase-activity inhibition and knockdown of PKG-Iα/β caused significant increases in apoptosis (i.e. decreases in cell survival) and dramatically decreased the cell proliferation, colony formation and cell attachment in both the lung cancer and mesothelioma cells.

## Conclusion

The mesothelioma cells in this study express both PKG-Iα and PKG-Iβ, and the inhibition of their expression or kinase activity results in dramatic suppression of the phosphorylation of c-Src at serine-17 and the endogenous activation of c-Src as well as the cell survival, proliferation and attachment of these cells. Because the kinase inhibitors and shRNA constructs are (at present) not able to discriminate between the two PKG-I isoforms, it is not yet possible to identify which PKG-I isoform is mediating the c-Src phosphorylation and the pro-survival, pro-growth and pro-attachment effects of PKG-I in mesothelioma cells. In the NCI-H23 lung cancer cells, which express only the PKG-Iα isoform, the PKG-Iα was identified as a key protein kinase mediating the enhanced phosphorylation and activation of c-Src and the downstream enhancement of cell survival (contributing to chemoresistance), proliferation and attachment of these lung cancer cells. This builds upon our previous observation that c-Src kinase activity promotes tyrosine-phosphorylation and activation of PKG-Iα in ovarian cancer cells. Thus, it appears that the interactions between c-Src and PKG-I in cancer cells may represent a novel “oncogenic re-enforcement”, with each protein kinase phosphorylating and enhancing the kinase activity of the other, ultimately contributing to chemoresistance and tumor growth. This interaction between c-Src and PKG-I may provide a novel target for the development of new anti-cancer therapeutic agents.
